# Left Ventricular Apical Intracardiac Mass With Embolic Stroke During Aggregatibacter segnis Bacteremia Following Acute Cholangitis: A Case Report

**DOI:** 10.7759/cureus.103020

**Published:** 2026-02-05

**Authors:** Yuki Chiko, Shizuma Omote

**Affiliations:** 1 Internal Medicine, Fukuyama Minami Hospital, Fukuyama, JPN

**Keywords:** aggregatibacter segnis, apical vegetation, hacek group, haemophilus segnis, infectious-septic endocarditis

## Abstract

An elderly woman with cholangiocarcinoma and a metallic biliary stent presented with fever and anorexia and was initially treated for acute cholangitis with biliary drainage and intravenous ampicillin/sulbactam. Two sets of blood cultures grew *Aggregatibacter segnis* (*A. segnis*)* *identified by matrix-assisted laser desorption/ionization-time-of-flight (MALDI-TOF) mass spectrometry, prompting evaluation for infective endocarditis (IE). Transthoracic echocardiography (TTE) showed no vegetation but revealed apical hypokinesis suggestive of Takotsubo cardiomyopathy. Despite antimicrobial therapy, fever persisted, and repeat TTE on hospital day 13 demonstrated a newly developed, highly mobile, irregular mass measuring 14 × 7 mm at the left ventricular apex. On hospital day 15, the patient developed aphasia and right-sided hemiparesis, and brain magnetic resonance imaging revealed extensive acute cerebral infarction involving the left hemisphere. Follow-up TTE performed immediately after the neurological event showed the complete disappearance of the apical mass, suggesting embolization. This case highlights the importance of careful echocardiographic follow-up in patients with *A. segnis* bacteremia, even when initial imaging is unremarkable, particularly in the presence of apical wall-motion abnormalities that may alter intracardiac flow.

## Introduction

The HACEK group, *Haemophilus parainfluenzae*, *Aggregatibacter* species (*A. actinomycetemcomitans*, *A. aphrophilus*, *A. paraphrophilus*, and *A. segnis*), *Cardiobacterium* species (*C. hominis* and *C. valvarum*), *Eikenella corrodens*, and *Kingella* species, is a well-recognized cause of infective endocarditis (IE) among gram-negative organisms [1]. These bacteria are nutritionally fastidious and slow growing, often making microbiological diagnosis challenging. HACEK organisms account for approximately 1%-3% of all IE cases, and reports of non-cardiac infections caused by these organisms remain limited [2]. *Aggregatibacter segnis* (formerly classified as *Haemophilus segnis*) was later reclassified into the genus *Aggregatibacter* [3]. We encountered an elderly patient with cholangiocarcinoma and a biliary stent who presented with fever and was initially treated for acute cholangitis, in whom *A. segnis* was isolated from blood cultures. Although initial transthoracic echocardiography (TTE) revealed no vegetation, follow-up examinations demonstrated the development of an apical wall-motion abnormality and a newly formed apical vegetation. To our knowledge, infective endocarditis associated with *A. segnis* occurring in the clinical context of acute cholangitis has not been previously reported. In addition, vegetation formation at a non-valvular site in association with apical wall-motion abnormalities is exceedingly rare. Non-valvular vegetations are clinically significant because they are uncommon, may be overlooked on initial echocardiography, and can be associated with altered intracardiac flow and an increased risk of systemic embolization. The awareness of such atypical locations is therefore important for timely diagnosis and follow-up.

## Case presentation

An elderly woman in her 80s with a medical history of gastroesophageal reflux disease and osteoporosis had undergone the placement of a metallic biliary stent for extrahepatic cholangiocarcinoma in October of the previous year and was subsequently managed with palliative care. She had been hospitalized in April of the current year for a liver abscess, which improved with antimicrobial therapy. In mid-June, she presented with fever and a loss of appetite. On arrival, her vital signs were as follows: temperature, 36.7°C; pulse, 85 beats/minute and regular; blood pressure, 116/83 mmHg; and oxygen saturation, 98% on room air. Her Glasgow Coma Scale score was 14 (E3V5M6). Physical examination revealed jaundice of the skin, pale palpebral conjunctiva, mild tenderness in the upper abdomen, and pitting edema of the lower extremities. Lung auscultation was clear, and heart sounds were regular without murmurs. No peripheral stigmata of infective endocarditis, including Janeway lesions or Osler nodes, were observed. No obvious periodontal disease or recent dental procedures were noted. Laboratory findings on admission showed predominantly direct hyperbilirubinemia, elevated hepatobiliary enzymes, and increased inflammatory markers (Table 1).

**Table 1:** Laboratory findings WBC, white blood cell count; Hb, hemoglobin; Hct, hematocrit; PLT, platelet count; T-Bil, total bilirubin; D-Bil, direct bilirubin; AST, aspartate transaminase; ALT, alanine transaminase; LDH, lactate dehydrogenase; γ-GTP, γ-guanosine triphosphate; BUN, blood urea nitrogen; Cr, creatinine; CRP, C-reactive protein; APTT, activated partial thromboplastin time; PT, prothrombin time; INR, international normalized ratio

	Patient value	Unit	Reference range
Hematology			
WBC	18970	/μL	3300-8600
Hb	9.2	g/dL	13.7-16.8
Hct	25.3	%	40.7-50.1
PLT	15.4	×10^4^/μL	15.8-34.8
Biochemistry			
T-Bil	13.0	mg/dL	0.4-1.5
D-Bil	8.4	mg/dL	0.1-0.6
AST	66	IU/L	13-30
ALT	51	IU/L	10-42
LDH	209	IU/L	124-222
γ-GTP	144	IU/L	13-64
BUN	11.1	mg/dL	8-20
Cr	0.41	mg/dL	0.65-1.07
Na	129	mEq/L	138-145
K	2.1	mEq/L	3.6-4.8
Cl	91	mEq/L	101-108
Ca	7.4	mg/dL	8.8-10.1
Serology			
CRP	17.79	mg/dL	0.00-0.14
Blood coagulation test			
APTT	40.4	Seconds	24.0-36.0
PT	12.2	Seconds	10.0-12.0
PT-INR	1.2		0.90-1.10

**Figure 1:**
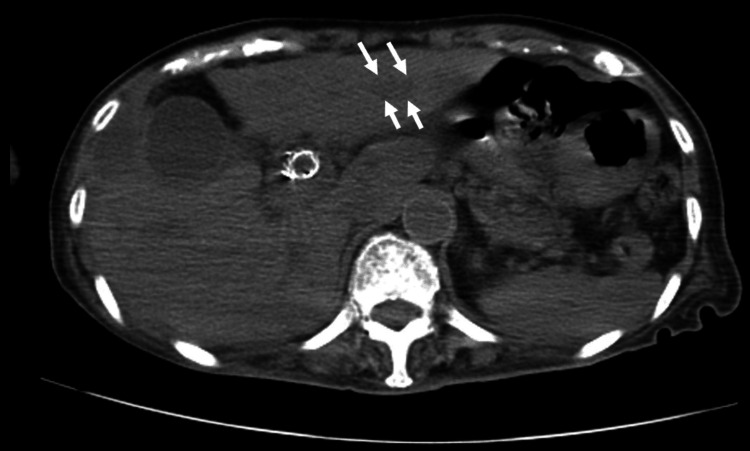
Non-contrast CT at admission Non-contrast abdominal computed tomography (CT) at admission showing the dilatation of the intrahepatic bile ducts (arrow)

Abdominal computed tomography demonstrated the progression of intrahepatic biliary dilatation compared to previous imaging, with a metallic stent in situ (Figure 1).

Based on these findings, acute cholangitis was suspected. Biliary drainage was performed on the day of admission, and empirical antimicrobial therapy with ampicillin/sulbactam was initiated. Two sets of blood cultures obtained at admission subsequently yielded gram-negative rods, which were identified as *A. segnis* by matrix-assisted laser desorption/ionization-time-of-flight (MALDI-TOF) mass spectrometry. Antimicrobial susceptibility testing was performed, and the results are shown in Table 2.

**Table 2:** Susceptibility results for bacteria ABPC, ampicillin; AMPC, amoxicillin; PIPC, piperacillin; CAZ, ceftazidime; CTRX, ceftriaxone; CFPM, cefepime; MEPM, meropenem; AZT, aztreonam; AMPC/CVA, amoxicillin/clavulanic acid; ABPC/SBT, ampicillin/sulbactam; PIPC/TAZ, piperacillin/tazobactam; AZM, azithromycin; MINO, minocycline; LVFX, levofloxacin; ST, sulfamethoxazole-trimethoprim; S, susceptible; MIC, minimum inhibitory concentration

*Aggregatibacter segnis*		
	MIC	
ABPC	≦1	S
AMPC	≦1	S
PIPC	≦1	S
CAZ	≦1	S
CTRX	≦1	S
CFPM	≦1	S
MEPM	≦0.5	S
AZT	≦1	S
AMPC/CVA	≦2	S
ABPC/SBT	0.25	S
PIPC/TAZ	≦0.25	S
AZM	2	S
MINO	≦2	S
LVFX	≦2	S
ST	≦10	S

**Figure 2:**
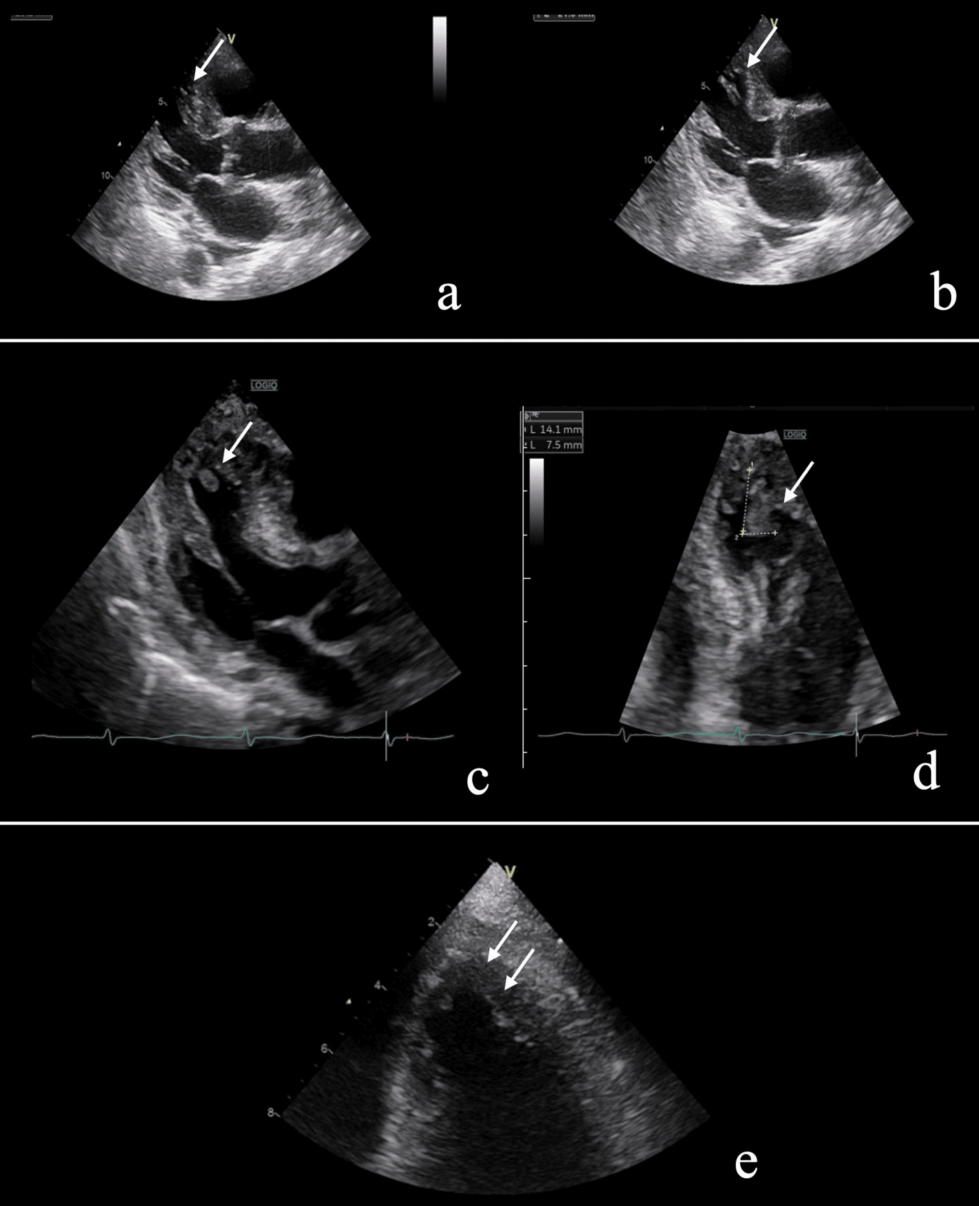
Transthoracic echocardiography (TTE) findings (a) Long-axis view during systole on initial examination. The apical region shows no systolic contraction (arrow), and no definite vegetations are identified. (b) Long-axis view on initial examination during diastole. The apex is the same as during systole (arrow). (c) Follow-up long-axis view on hospital day 13 demonstrating a newly developed apical vegetation on long-axis view (arrow). (d) Details of apical vegetation on hospital day 13 (arrow). It measures 14 × 7 mm, is highly mobile, and has an irregular surface. (e) On TTE performed immediately after the cerebral infarction was identified, the vegetation had completely disappeared (arrow)

Given the possibility of polymicrobial biliary infection, ampicillin/sulbactam therapy was continued. Because *A. segnis* is a known cause of infective endocarditis, transthoracic echocardiography (TTE) was performed after the identification of the organism. Although no vegetation was detected, TTE revealed apical wall-motion abnormality characterized by apical hypokinesis, suggestive of Takotsubo cardiomyopathy (Figure 2a).

Despite appropriate antimicrobial therapy and biliary drainage, the patient’s fever persisted.

**Figure 3:**
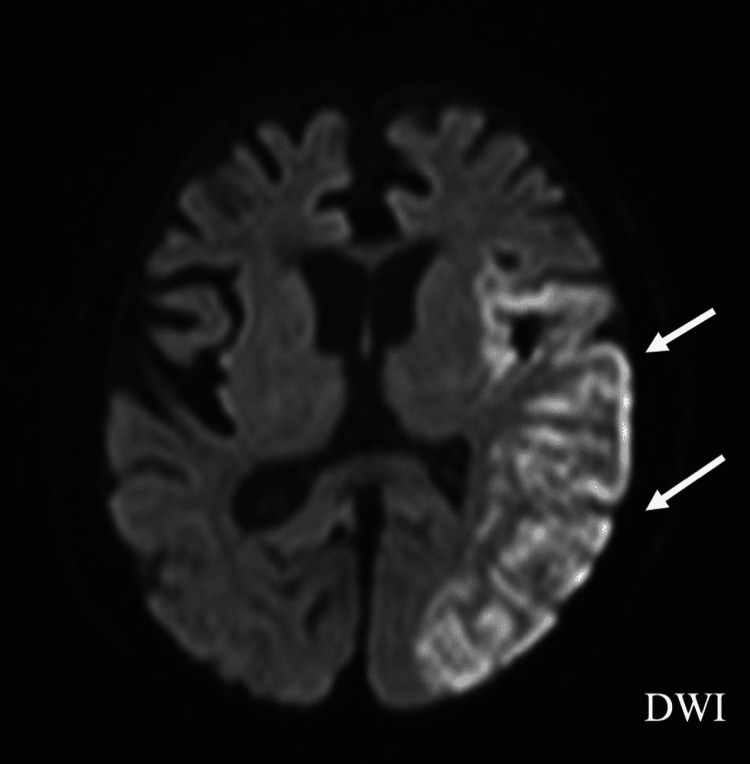
Diffusion-weighted MRI Diffusion-weighted brain magnetic resonance imaging (MRI) demonstrating extensive acute cerebral infarction involving the left hemisphere (arrow) DWI: diffusion-weighted imaging

Despite biliary drainage and appropriate antimicrobial therapy, inflammatory markers partially improved compared to admission levels but remained persistently elevated at approximately 5 mg/dL of C-reactive protein, accompanied by intermittent fever. On hospital day 13, repeat TTE demonstrated a newly developed, highly mobile, irregular mass measuring 14 × 7 mm at the left ventricular apex (Figure 2b). Based on the presence of bacteremia, the newly detected intracardiac mass, and subsequent vascular events, the clinical course was considered consistent with infective endocarditis according to the 2023 Duke-International Society for Cardiovascular Infectious Diseases (ISCVID) criteria [4]. On hospital day 15, the patient developed aphasia and right-sided hemiparesis. Brain magnetic resonance imaging revealed extensive acute cerebral infarction involving the left hemisphere (Figure 3).

Follow-up TTE performed immediately after the neurological event demonstrated the complete disappearance of the apical mass (Figure 2c), suggesting embolization. The patient ultimately completed a total of six weeks of antimicrobial therapy based on the clinical diagnosis of infective endocarditis. Given the patient’s advanced age, underlying malignancy, and functional status, the decision was made to continue conservative management in accordance with the wishes of the patient’s family. Although no long-term functional follow-up could be obtained, the patient completed a total of six weeks of antimicrobial therapy; however, her general condition gradually deteriorated, and she ultimately died.

## Discussion

*Aggregatibacter segnis*, a member of the HACEK group, is a nutritionally fastidious gram-negative coccobacillus that is part of the normal human oropharyngeal flora [5]. Although HACEK organisms are well-recognized as causative pathogens of infective endocarditis (IE), reports of non-cardiac infections caused by these organisms remain limited. In particular, *A. segnis* is an uncommon pathogen in clinical practice, and only a small number of cases of *A. segnis*-associated IE have been reported to date [5,6]. In the present case, *A. segnis* was isolated from blood cultures obtained at the time of presentation for suspected acute cholangitis in a patient with a biliary stent placed for cholangiocarcinoma. Although bile cultures were not obtained, the clinical presentation and radiological findings were consistent with acute cholangitis, making the biliary tract a plausible source of bacteremia. However, the involvement of other biliary pathogens cannot be completely excluded, and the precise portal of entry for *A. segnis* remains uncertain.

HACEK-associated IE is traditionally thought to originate from the oral cavity, often in association with poor oral hygiene or recent dental procedures [2]. In the present patient, no obvious periodontal disease or recent dental interventions were identified. By contrast, previous studies have demonstrated that biliary stents in patients with malignancy can become colonized by microorganisms, including those originating from the oral cavity [7,8]. These findings raise the possibility that *A. segnis* colonized the biliary stent and subsequently entered the bloodstream during the episode of cholangitis. While this hypothesis is biologically plausible, microbiological confirmation from bile or stent cultures was not available in this case.

Another notable aspect of this case is the development of an intracardiac mass at the left ventricular apex rather than on a cardiac valve. Vegetations in IE typically form on valves at sites of endothelial injury, and the involvement of non-valvular regions is exceedingly rare [9]. In this patient, initial transthoracic echocardiography demonstrated an apical wall-motion abnormality consistent with Takotsubo cardiomyopathy. Takotsubo cardiomyopathy is characterized by transient apical akinesis and is known to predispose patients to left ventricular thrombus formation due to altered intracardiac flow and blood stasis [10-12]. Accordingly, an important differential diagnosis of the apical mass in this case was left ventricular thrombus. However, several findings favored an infectious vegetation rather than a bland thrombus. These included sustained bacteremia due to a HACEK organism, persistent inflammatory activity despite appropriate antimicrobial therapy, the highly mobile and irregular morphology of the mass observed on echocardiography, and its rapid disappearance following the development of an embolic cerebral infarction.

Although follow-up blood cultures obtained after the initiation of antimicrobial therapy were negative, the appearance of a new intracardiac mass on serial echocardiography and the subsequent embolic event strongly supported the clinical diagnosis of infective endocarditis. Anticoagulation was considered as a potential management option if the intracardiac mass had been interpreted as a left ventricular thrombus. However, given the strong suspicion of infective endocarditis and concern for septic embolization, anticoagulation was not initiated. In addition, the patient’s advanced age, underlying malignancy, and overall clinical condition influenced the decision to prioritize conservative management.

According to the 2023 Duke-ISCVID criteria, the combination of sustained bacteremia due to a HACEK organism, the appearance of a newly developed intracardiac mass on follow-up echocardiography, and the occurrence of embolic cerebral infarction was highly suggestive of IE [4]. Although the mass was detected at a non-valvular site and pathological confirmation was not available, the temporal relationship between the echocardiographic findings and the embolic event supported the clinical diagnosis of IE with embolization. Given the presence of underlying malignancy, Trousseau syndrome was considered in the differential diagnosis of cerebral infarction; however, the appearance of the intracardiac mass on serial echocardiography and its subsequent disappearance after the neurological event favored an intracardiac source of embolism. This case underscores the importance of careful clinical and echocardiographic monitoring in patients with persistent bacteremia due to rare pathogens such as *A. segnis*. Even when initial echocardiography does not reveal vegetation, repeated imaging may be warranted, particularly in patients with apical wall-motion abnormalities or other conditions that may alter intracardiac hemodynamics.

## Conclusions

This case suggests that *Aggregatibacter segnis* bacteremia associated with biliary tract disease may be followed by infective endocarditis. Careful echocardiographic follow-up should be considered, particularly when apical wall-motion abnormalities are present.
